# Случай тестикулярного нарушения формирования пола при кариотипе 46,XX, обусловленный мнимым синонимичным вариантом в гене <i>WT1</i>: трудности дифференциальной диагностики синдрома внутриутробной вирилизации у девочки

**DOI:** 10.14341/probl13436

**Published:** 2024-04-02

**Authors:** А. А. Буянова, И. Г. Воронцова, А. Ф. Самитова, Ю. А. Василиадис, Е. Е. Петряйкина, Е. С. Демина, А. Н. Тюльпаков

**Affiliations:** Центр высокоточного редактирования и генетических технологий для биомедицины; Российская детская клиническая больница; Центр высокоточного редактирования и генетических технологий для биомедицины; Центр высокоточного редактирования и генетических технологий для биомедицины; Российская детская клиническая больница; Российская детская клиническая больница; Российская детская клиническая больница; Медико-генетический научный центр им. акад. Н.П. Бочкова

**Keywords:** нарушения формирования пола, вирилизация, WT1, синдром де ля Шапеля, 46, XX тестикулярное нарушение формирования пола

## Abstract

Термин нарушения формирования пола (НФП) объединяет группу врожденных состояний, при которых имеет место несоответствие между хромосомным и (или) гонадным полом и строением половых органов. К одной из групп НФП относятся тестикулярные нарушения при кариотипе 46,XX (ТНФП_46,XX), в структуре которой выделяют формы, обусловленные транслокацией гена SRY, и более редко — SRY-негативные формы. В настоящем сообщении нами представлено наблюдение пациента с SRY-негативным ТНФП_46,XX, у которого первоначально состояние расценивалось как вирильная форма врожденной дисфункции коры надпочечников (ВДКН), затем — как идиопатическая внутриутробная вирилизация у девочки. На фоне вирилизации в возрасте 11 лет было заподозрено наличие тестикулярной ткани. При молекулярно-генетическом обследовании (полноэкзомное секвенирование с валидацией методом С энгера) был обнаружен de novo вариант в экзоне 9 гена WT1 (chr11:32413528T>C), который по предсказаниям не приводил к изменению аминокислотной последовательности (p.Thr479=, NM_024426.6), однако нарушал сплайсинг, результатом чего было характерное для ТНФП_46,XX изменение C-концевого домена WT1. После верификации диагноза проведена гонадэктомия и назначена заместительная терапия эстрогенами. Таким образом, нами описан пациент с редкой формой ТНФП_46,XX, обусловленного вариантом в гене WT1. Представленное наблюдение иллюстрирует сложности дифференциальной диагностики синдрома внутриутробной вирилизации при женском кариотипе.

## Abstract (en)

Disorders of sex development (DSD) represent a group of congenital conditions in which there is a discrepancy between the chromosomal and (or) gonadal sex and the structure of the genitals. Within the DSD there is a subgroup of 46,XX testicular DSD (46,XX TDSD), which may be caused by the translocation of the SRY gene, and more rarely — due to other causes (SRY-negative forms). In this report, we present an observation of a patient with SRY-negative 46,XX TDSD, in whom the condition was initially regarded as a virile form of congenital adrenal hyperplasia, then as idiopathic intrauterine virilization in a girl. Due to the development of virilization at the age of 11, the presence of testicular tissue was suspected. Molecular genetic analysis (whole exome sequencing with Sanger validation) revealed a de novo variant in exon 9 of the WT1 gene (chr11:32413528T>C), which, according to predictions, did not lead to a change in the amino acid sequence (p.Thr479=, NM_024426.6), but disrupted splicing, resulting in a previously described in 46,XX TDSD a change in the C-terminal domain of WT1. After verification of the diagnosis, a gonadectomy was performed and estrogen replacement therapy was prescribed. Thus, we have described a patient with a rare form of 46,XX TDSD caused by a variant in the WT1 gene. The presented observation illustrates the difficulties of differential diagnosis of intrauterine virilization syndrome in female karyotype.

## АКТУАЛЬНОСТЬ

Термин нарушения формирования пола (НФП) объединяет группу врожденных состояний, при которых имеет место несоответствие между хромосомным и (или) гонадным полом и строением половых органов [1]. У млекопитающих дифференцировка пола в период внутриутробного развития является чрезвычайно сложным процессом, затрагивающим поэтапное включение разнообразных транскрипционных факторов, оказывающих как активирующее, так и супрессирующее воздействие. У человека до 6-й недели гестации гонада бипотенциальна, и ее дальнейшая дифференцировка в яичко или яичник зависит от хромосомного пола и происходит на фоне конкурентного взаимодействия так называемых протестикулярных и проовариальных факторов [2]. Ведущая роль в дифференцировке яичка принадлежит транскрипционному фактору SRY. Данный белок, кодируемый одноименным геном на коротком плече Y-хромосомы в локусе Yp11.2, активирует транскрипцию генов группы SOXE, прежде всего SOX9, что запускает процессы, инициирующие формирование яичка, а также оказывает репрессивное воздействие на проовариальное развитие [3]. В отсутствие SRY активируются несколько сигнальных путей с участием проовариальных генов, таких как WNT4/RSPO1, FOXL2 и RUNX1 [3].

Следует отметить, что наличие кариотипа 46,XX полностью не исключает закладку тестикулярной ткани. В структуре НФП 46,XX выделяют группу тестикулярных и овотестикулярных нарушений, когда при «женском» кариотипе происходит дифференцировка гонад в яичко или овотестис соответственно [4]. Нередко при таких состояниях выявляется транслокация гена SRY, как правило на хромосому X, однако у части пациентов ген SRY отсутствует. Механизм закладки яичка при SRY-негативных формах тестикулярного НФП 46,XX (ТНФП_46,XX) не всегда очевиден. В ряде случаев он может быть объяснен повышенной экспрессией генов, которые в норме активируются SRY, что наблюдается, например, при дупликациях в регуляторной области SOX9 [5]. Между тем причиной ТНФП_46,XX могут быть и патогенные варианты в других генах, отличных от генов группы SOXE. К их числу относятся описанные недавно варианты, затрагивающие консервативный C-концевой домен транскрипционного фактора WT1 [6][7].

Нами представлено описание клинического случая, первоначально расцениваемого как вирильная форма ВДКН, а затем как идиопатическая внутриутробная вирилизация у девочки. Диагноз был уточнен при проведении полноэкзомного секвенирования, при котором был выявлен вариант в гене WT1 с типичной локализацией для ТНФП_46,XX.

## ОПИСАНИЕ КЛИНИЧЕСКОГО СЛУЧАЯ

Ребенок от третьей беременности. Роды 2-е срочные, вес — 3460 г, рост — 53 см. Неправильное строение наружных гениталий выявлено при рождении. На 7-е сутки переведена в стационар для обследования, где на основании проявлений вирилизации, данных кариотипа (46,XX) и повышения уровня тестостерона (4,9 нмоль/л) установлен диагноз: ВДКН, вирильная форма. Однако, учитывая отсутствие электролитных нарушений и нормальный уровень 17OHP в крови (при скрининге — 3,8 нмоль/л, ретест — 8,6 нмоль/л), от терапии глюкокортикоидами было решено воздержаться.

При стационарном обследовании в возрасте 10 мес была констатирована III ст. вирилизации по Прадеру. ДНК-анализ на ген SRY был отрицательный. При гормональном обследовании: АКТГ — 16,6 пг/мл (0–46), 17OHP — 0,7 нмоль/л (0,1–2,9), ренин — 5,1 нг/мл/ч (1,9–6,0), кортизол — 159 нмоль/л (130–640), ЛГ — 0,3 Ед/л (0,1–3,9), ФСГ — 4,7 Ед/л (0,6–6,1), тестостерон базальный <0,35 нмоль/л (0,1–0,4), тестостерон на 3-дневной пробе с хорионическим гонадотропином (ХГ) — 3,7 нмоль/л. Полученные результаты позволили исключить ВДКН. Предварительный диагноз: «Дисгенезия гонад? Идиопатическая внутриутробная вирилизация?» В возрасте 1,5 года выполнен 1-й этап феминизирующей пластики. В последующем наблюдалась амбулаторно. При контрольных стационарных обследованиях состояние без существенной динамики.

Повторное обращение в возрасте 11 лет 9 мес в связи с жалобами на увеличение клитора, огрубление голоса. При поступлении: рост — 150 см (SDS роста: 0,35), вес — 44 кг (SDS ИМТ: +0,63). В соматическом статусе без особенностей. Половое развитие по Таннеру B1P2. Наружные гениталии: гипертрофия головки клитора, половые губы увеличены в размерах, мошонкообразные, hymen не эстрогенизирован, не гиперемирован, визуализируется вход во влагалище, сформированный оперативным путем. Костный возраст: соответствует 11,5 года. При УЗИ органов малого таза определены матка (27*8*13 мм) и яичники (правый 15*10 мм, левый 14*8 мм) без выраженного фолликулярного аппарата. При гормональном обследовании: ЛГ — 8,92 МЕ/л, (0–4,3), ФСГ — 40,07 МЕ/л (0,3–7,8), эстрадиол <36,70 пмоль/л (0–345), тестостерон — 12,76 нмоль/л (0–0,98), дегидроэпиандростерон-сульфат — 0,84 мкмоль/л (0,9–7,3), бета-ХГЧ — 0,24 МЕ/л (0–4,7), СА-125 — 8,96 Ед/ мл (0–35), альфафетопротеин — 0,54 Мед/мл (0–7,29). В качестве вероятной причины внутриутробной вирилизации рассматривался дефицит ароматазы. Для оценки состояния гипоталамо-гипофизарно-гонадной системы пациенту была назначена пробная терапия эстрогенами — эстрадиола валерат per os в дозе 1,0 мг — 1 нед., затем 2,0 мг — 1 нед. Гормональные показатели через 2 нед. терапии эстрогенами: ЛГ — 3,4 МЕ/л, ФСГ — 16,4 МЕ/л, эстрадиол — 139,7 пмоль/л, тестостерон — 1,7 нмоль/л. Динамика гормональных показателей свидетельствовала о сохранной регуляции гипоталамо-гипофизарно-гонадной системы в ответ на лечение эстрогенами.

Для уточнения диагноза проведена молекулярно-генетическая диагностика. После получения информированного согласия родителей произведено выделение геномной ДНК из образца периферической крови пациента с использованием наборов Qiagen. Пробоподготовка включала ультразвуковую фрагментацию геномной ДНК (Covaris S220) и обогащение библиотек фрагментов ДНК последовательностями экзонов с помощью зондов Agilent All Exon v8 по лабораторному протоколу [8]. Секвенирование белок-кодирующих последовательностей осуществлялось методом парно-концевых прочтений на приборе G-400 (MGI Tech) по протоколу производителя. Для анализа данных использован автоматизированный пайплайн, написанный на языке python3, включающий в себя несколько этапов. Оценку качества секвенирования проводили программой FastQC v0.11.9, разбалансированные основания в начале прочтений были удалены программой BBDuk v38.96. Выравнивание прочтений на референсную сборку генома человека GRCh37/hg19 осуществлялось при помощи bwa-mem2 v2.2.1. Дубликаты были маркированы программой Picard v2.22.4 и исключены из дальнейшего анализа. Коллинг вариантов осуществлялся с помощью bcftools mpileup v1.9 и Strelka2 v2.9.2 с получением файлов формата vcf. Они были нормализованы программой vt normalize v0.5772 и отфильтрованы по таргетным регионам, расширенным на ± 100 пар оснований с каждого конца. Метрики покрытия были получены программой Picard v2.22.4. Аннотация полученных вариантов была реализована при помощи ANNOVAR. Анализ числа копий (инсерций и делеций) проводился с помощью CNVkit, аннотация полученных вариантов числа копий в соответствии с критериями ACMG [9]. Интерпретация клинической значимости выявленных вариантов производилась в соответствии с критериями ACMG [10][11] с использованием баз данных вариантов и литературных источников. В результате ДНК-анализа у пациента обнаружен вариант нуклеотидной последовательности в экзоне 9 гена WT1 (chr11:32413528T>C) в гетерозиготном состоянии (p.Thr479=, NM_024426.6).

Праймеры, необходимые для валидации гена WT1 у родителей, а также для подтверждения найденного варианта у пациента с помощью секвенирования по Сэнгеру, были разработаны с использованием Primer3Plus. ПЦР проводили в конечном объеме 25 мкл с использованием 50x Tersus полимеразы (Evrogen), 0,5 мкл 50x dNTP, по 1 мкл каждого праймера с концентрацией 10 мкМ и 50 нг геномной ДНК. Полученные ампликоны длиной 582 п.н. очищали с помощью магнитных частиц KAPA HyperPure Beads (Roche). Секвенирование образцов методом Сэнгера проводилось на генетическом анализаторе 3500xL Applied Biosystems в компании «Евроген».

Полученные результаты подтвердили отсутствие мутации у родителей и позволили установить диагноз ТНФП_XX (рис. 1).

**Figure:**
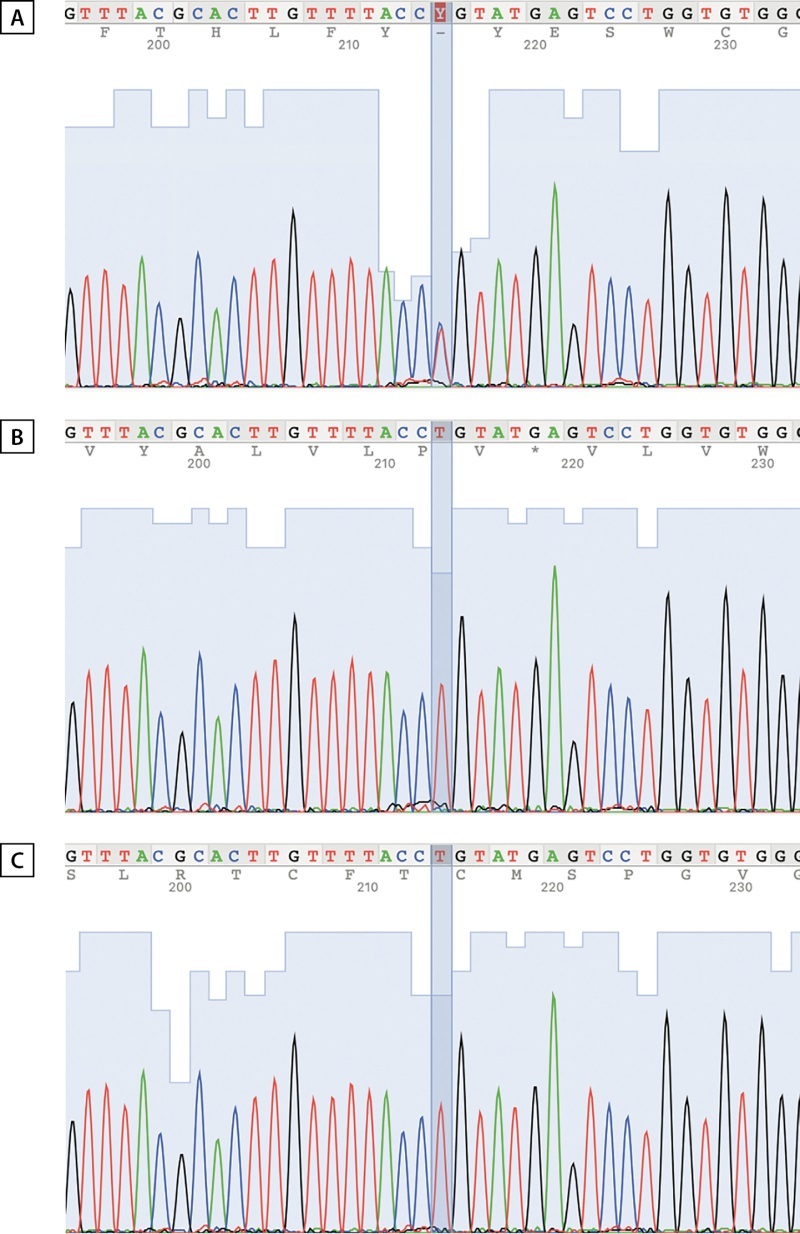
Рисунок 1. Сравнение хроматограмм последовательностей.A — пациент, B — отец пациента, C — мать пациента.

В возрасте 11 лет 10 мес пациентке проведена операция. При лапароскопии тело матки определено по средней линии, в виде соединительнотканного тяжа, маточные трубы не определяются, в латеральных каналах за подвздошными сосудами с обеих сторон визуализированы гонады, правая — 20*10*22 мм, левая — 28*15*25 мм, серой окраски, овальной формы, поверхность гладкая, с окружающими тканями не спаяна. Произведена двусторонняя гонадэктомия. При гистологическом исследовании определена ткань гонад, представленная многочисленными тесно расположенными семенными канальцами, в которых определяются клетки Сертоли, некоторые канальцы атрофированы, гиалинизированы, между канальцами определяются клетки Лейдига. Пациентка выписана на терапии эстрогенами — эстрадиола валерат 0,5 мг/сут с последующим повышением дозы.

## ОБСУЖДЕНИЕ

Представленное нами наблюдение иллюстрирует сложности дифференциальной диагностики НФП. В классификации моногенных форм НФП, предложенной европейскими экспертами, выделяют состояния с избытком андрогенов при женском кариотипе (46,XX DSD with androgen excess) [4], к которым относятся несколько форм ВДКН (гены CYP21A2, CYP11B1, HSD3B2, POR), резистентность к глюкокортикоидам (NR3C1), резистентность к эстрогенам (ESR1) и дефицит ароматазы (CYP19A1). ВДКН является, безусловно, самой частой причиной внутриутробной вирилизации у девочек, однако отсутствие повышения 17OHP в крови при нормальных уровнях кортизола и АКТГ позволили исключить данный диагноз, в том числе его редкие формы. Вирилизация и повышение уровня андрогенов при резистентности к глюкокортикоидам сопровождаются также повышением концентраций кортизола и АКТГ, что не отмечалось при обследовании пациента. Дефект рецептора к эстрогенам (ESR1) был также исключен на основании низкого уровня эстрадиола. Что касается дефицита ароматазы, то данный диагноз рассматривался нами как вероятный. В пользу дефицита CYP19A1 были внутриутробная вирилизация без прогрессии после рождения, повторное повышение уровня андрогенов в период пубертата, неопределяемые концентрации эстрадиола в пубертате и эстроген-зависимое снижение уровней тестостерона и гонадотропинов. Между тем некоторые признаки не укладывались в дефицит ароматазы, прежде всего — отсутствие увеличенных поликистозных яичников и незначительное повышение уровней гонадотропинов. Дифференциально-диагностический поиск был завершен при проведении полноэкзомного секвенирования — выявлен патогенный вариант в гене WT1, ассоциированный с ТНФП_46,XX.

Ген WT1 был локализован на коротком плече 11-й хромосомы в участке 11p13 в процессе картирования региона, делецированного при опухоли Вильмса (Wilms tumor) [12]. Белок WT1 имеет 4 основные изоформы, насчитывающие от 502 до 522 аминокислотных остатков, и представляет собой содержащий цинковые пальцы ДНК-связывающий белок (zinc finger protein), функционально являющийся транскрипционным активатором или репрессором в зависимости от тканевого или хромосомного контекста [13]. WT1 играет важную роль в процессе эмбрионального развития мочеполовой системы и мезотелиальных тканей [14], и нарушения его функции ассоциированы с широким спектром врожденной патологии почек и НФП. Герминальные мутации в гене WT1 описаны при опухоли Вильмса, тип 1 [15], синдроме Дениса-Драша (сочетание опухоли Вильмса, НФП 46,XY и гломерулонефрита) [16], синдроме Фрезье (сочетание дисгенезии гонад при кариотипе 46,XY и гломерулонефрита) [17], нефротическом синдроме, тип 4 [18], и синдроме Мичем (НФП 46,XY, врожденная диафрагмальная грыжа и возможное сочетание с удвоением влагалища, пороком сердца и легких) [19].

Патогенные варианты в гене WT1 как причина ТНФП_46,XX были описаны совсем недавно. Eozenou с соавт. при обследовании 78 пациентов с SRY-негативными тестикулярными или овотестикулярными НФП при кариотипе 46,XX выявили 7 случаев заболевания, обусловленных патогенными de novo вариантами в гене WT1 [6]. Из 4 пациентов с ТНФП_46,XX у 3 была IV степень вирилизации по Прадеру, и гонады (дисгенетичные яички) в брюшной полости, тогда как еще в 1 случае было правильное мужское строение наружных гениталий, и яички определялись в мошонке [6]. Примечательно, что все выявленные авторами патогенные варианты в гене WT1 затрагивали последовательность последнего 10-го экзона, что приводило (как было доказано in vitro), к нарушению структурной стабильности 4-го цинкового пальца (ZF4) белка WT1 [6]. Полученные экспериментальные данные позволяют предполагать, что такие нарушения функции ZF4 приводят к относительной активации сигналинга с участием протестикулярных транскрипционных факторов (в сравнении с проовариальными), что и объясняет формирование фенотипа ТНФП_46,XX в процессе внутриутробного развития [6].

Обнаруженный нами в ходе диагностического поиска вариант WT1(NM_024426.6): c.1437A>G был ранее описан Sirokha с соавт., которые диагностировали ТНФП_46,XX у пациента с двойственным строением наружных гениталий (2-я стадия по Прадеру) и двусторонним крипторхизмом [7]. Как и в представленном нами наблюдении, авторами было отмечено повышение тестостерона в ответ на стимуляцию ХГ. Особенностью замены c.1437A>G является то, что такой вариант аннотируется при первичном биоинформатическом анализе как синонимичный p.Thr479= (кодоны ACA и ACG в положении 479 соответствуют треонину) и, как следствие, может интерпретироваться как доброкачественный. Между тем геномная позиция hg19_chr11:32413528 соответствует предпоследнему нуклеотиду одного из альтернативных экзонов гена WT1, и замена c.1437A>G приводит к нарушению консервативного донорного сайта сплайсинга, результатом чего, как было доказано in vitro [7], является удержание интрона 9 в молекуле мРНК и потеря последовательности цинкового пальца ZF4, которая в норме кодируется экзоном 10. Можно отметить, что молекулярный патогенез в результате замены c.1437A>G (дисфункция ZF4) такой же, как и при описанных ранее вариантах, ассоциированных с ТНФП_46,XX [6].

## ЗАКЛЮЧЕНИЕ

Таким образом, впервые в отечественной литературе нами описан случай ТНФП_46 XX, обусловленного вариантом в гене WT1. Представленное наблюдение иллюстрирует сложности дифференциальной диагностики синдрома внутриутробной вирилизации при женском кариотипе, проведение которой требует исключения ТНФП_46,XX, в том числе SRY-негативных форм. Выявленная нами однонуклеотидная замена в гене WT1 подчеркивает также необходимость более пристального внимания к т.н. доброкачественным вариантам (в т.ч. синонимичным), которые не встречаются или встречаются крайне редко в референсных базах данных как казуативные. Часть из них может оказывать влияние на сплайсинг, что должно учитываться при выборе соответствующих программных модулей в процессе проведения биоинформатического анализа.

## ДОПОЛНИТЕЛЬНАЯ ИНФОРМАЦИЯ

Источники финансирования. Работа выполнена по инициативе авторов без привлечения финансирования.

Конфликт интересов. Авторы декларируют отсутствие явных и потенциальных конфликтов интересов, связанных с содержанием настоящей статьи.

Участие авторов. Все авторы одобрили финальную версию статьи перед публикацией, выразили согласие нести ответственность за все аспекты работы, подразумевающую надлежащее изучение и решение вопросов, связанных с точностью или добросовестностью любой части работы.

Согласие пациента. Пациент добровольно подписал информированное согласие на публикацию персональной медицинской информации в обезличенной форме в журнале «Проблемы эндокринологии».
